# Supplementary material for: Attentional state, not trait, predicts test performance in video-based learning
Source: iScience. 2025 Sep 22;28(10):113622. doi: 10.1016/j.isci.2025.113622 (PMC12538096; doi:10.1016/j.isci.2025.113622)
Supplement: Document S1. Data S1 [file mmc1.pdf]

iScience, Volume *28*

Supplemental information

Attentional state, not trait, predicts test performance in video-based learning

Jens Madsen and Lucas C. Parra

Supplemental information

Data S1: Generalized linear model output

Factors affecting performance (score):

Generalized linear mixed-effects model fit by PL

Model information:

**Number of observations**	119
**Fixed effects coefficients**	10
**Random effects coefficients**	4
**Covariance parameters**	1
**Distribution**	Binomial
**Link**	Logit
**FitMethod**	MPL

Model Formula

Outcome:
Score

Fixed Effects:
GPA_{norm} + WMC + Inattentive + Hyperactive + ISC_{eeg-1} + ISC_{eeg-2} + ISC_{eeg-3} + ISC_{eeg-4} + ISC_{eeg-5}

Random Effects:
(1 | Exp)

Full Model Formula:
Score ~ 1 + GPA_{norm} + WMC + Inattentive + Hyperactive + ISC_{eeg-1} + ISC_{eeg-2} + ISC_{eeg-3} + ISC_{eeg-4} + ISC_{eeg-5} + (1 | Exp)

Model fit statistics:

AIC	BIC	LogLikelihood	Deviance
226.93	257.5	-102.47	204.93

Fixed effects coefficients (95% CIs):

Predictor	Estimate	SE	t-Stat	DF	p-Value	CI Lower	CI Upper
(Intercept)	-1.8019	0.32212	-5.5939	109	1.67E-07	-2.4404	-1.1635
GPA	0.20405	0.059833	3.4103	109	0.000911	0.085458	0.32263
WMC	0.067786	0.02009	3.374	109	0.001027	0.027967	0.1076
Inattentive	0.005509	0.006586	0.83649	109	0.40471	-0.00754	0.018562
Hyperactive	-0.00369	0.007807	-0.47325	109	0.63698	-0.01917	0.011779
ISC_{eeg-1}	19.474	4.7377	4.1105	109	7.67E-05	10.084	28.864
ISC_{eeg-2}	2.3485	6.9659	0.33714	109	0.73666	-11.458	16.155
ISC_{eeg-3}	11.688	11.633	1.0047	109	0.31728	-11.369	34.745
ISC_{eeg-4}	5.1097	13.017	0.39254	109	0.69542	-20.689	30.909
ISC_{eeg-5}	-4.8603	12.542	-0.38753	109	0.69912	-29.718	19.997

Random effects covariance parameters:

Group	Name1	Name2	Type	Estimate
Exp (4 Levels)	(Intercept)	(Intercept)	std	0.31245

Group	Name	Estimate
Error	sqrt(Dispersion)	1

Factors affecting ISC:

Generalized linear mixed-effects model fit by PL

Model information:

**Number of observations**	146
**Fixed effects coefficients**	4
**Random effects coefficients**	4
**Covariance parameters**	2
**Distribution**	Normal
**Link**	Identity
**FitMethod**	MPL

Model Formula

Outcome:

ISC_{eeg-1}

Fixed Effects:

1 + WMC + Inattentive + Hyperactive

Random Effects:

(1 | Exp)

Full Model Formula:

ISC_{eeg-1} ~ 1 + WMC + Inattentive + Hyperactive + (1 | Exp)

Model fit statistics:

AIC	BIC	LogLikelihood	Deviance
-932.31	-914.41	472.16	-944.31

Fixed effects coefficients (95% CIs):

Predictor	Estimate	SE	t-Stat	DF	p-Value	CI Lower	CI Upper
(Intercept)	0.022575	0.0046203	4.8861	142	2.74e-06	0.013442	0.031709
WMC	0.0012598	0.0004655	2.7067	142	0.00763	0.0003397	0.00218
Inattentive	0.0001427	0.0001695	0.8419	142	0.40129	-0.0001924	0.0004777
Hyperactive	0.0001129	0.0001971	0.5726	142	0.56782	-0.0002768	0.0005025

Random effects covariance parameters:

Group	Name1	Name2	Type	Estimate
Exp (4 Levels)	(Intercept)	(Intercept)	std	0.0052881

Group	Name	Estimate
Error	sqrt(Dispersion)	0.0092081

Factors affecting GPA

Generalized linear mixed-effects model fit by PL

Model information:

**Number of observations**	119
**Fixed effects coefficients**	4
**Random effects coefficients**	4
**Covariance parameters**	2
**Distribution**	Normal
**Link**	Logit
**FitMethod**	MPL

Model Formula

Outcome:

GPA_{norm}

Fixed Effects:

1 + WMC + Inattentive + Hyperactive

Random Effects:

(1 | Exp)

Full Model Formula:

GPA_{norm} ~ 1 + WMC + Inattentive + Hyperactive + (1 | Exp)

Model fit statistics:

AIC	BIC	LogLikelihood	Deviance
316.2	332.87	-152.1	304.2

Fixed effects coefficients (95% CIs):

Predictor	Estimate	SE	t-Stat	DF	p-Value	CI Lower	CI Upper
(Intercept)	1.291400	0.44894	2.8764	115	0.00479	0.40208	2.1806
WMC	0.083428	0.05040	1.6553	115	0.10058	-0.01640	0.18326
Inattentive	-0.034875	0.01630	-2.1396	115	0.03450	-0.06716	-0.00259
Hyperactive	0.031835	0.02144	1.485	115	0.14027	-0.01063	0.07430

Random effects covariance parameters:

Group	Name1	Name2	Type	Estimate
Exp (4 Levels)	(Intercept)	(Intercept)	std	0.23505

Group	Name	Estimate
Error	sqrt(Dispersion)	0.11232
